# Nanocomplex of Berberine with C_60_ Fullerene Is a Potent Suppressor of Lewis Lung Carcinoma Cells Invasion In Vitro and Metastatic Activity In Vivo

**DOI:** 10.3390/ma14206114

**Published:** 2021-10-15

**Authors:** Iryna Horak, Svitlana Prylutska, Iryna Krysiuk, Serhii Luhovskyi, Oleksii Hrabovsky, Nina Tverdokhleb, Daria Franskevych, Dmytro Rumiantsev, Anton Senenko, Maxim Evstigneev, Liudmyla Drobot, Olga Matyshevska, Uwe Ritter, Jacek Piosik, Yuriy Prylutskyy

**Affiliations:** 1 Palladin Institute of Biochemistry, NAS of Ukraine, 9 Leontovicha Str., 01030 Kyiv, Ukraine; iryna.horak@gmail.com (I.H.); iryna.kr@biochem.kiev.ua (I.K.); grabalexey@gmail.com (O.H.); drobot@biochem.kiev.ua (L.D.); matysh@yahoo.com (O.M.); 2 Faculty of Plant Ptotection, Biotechnology and Ecology, National University of Life and Environmental Science of Ukraine, 15 Heroiv Oborony Str., 03041 Kyiv, Ukraine; 3 Chebotarov Institute of Gerontology, NAS of Ukraine, 67 Vyshgorodska Str., 04114 Kyiv, Ukraine; lugsp61@gmail.com; 4 Leibniz Institute of Polymer Research Dresden, 6 Hohe Str., 01069 Dresden, Germany; tverdokhlebnm@gmail.com; 5 Department of Biophysics and Medical Informatics, Taras Shevchenko National University of Kyiv, 64 Volodymyrska Str., 01601 Kyiv, Ukraine; dashaqq@gmail.com (D.F.); prylut@ukr.net (Y.P.); 6 Institute of Physics, NAS of Ukraine, 46 Nauky Ave., 03028 Kyiv, Ukraine; rumiantsevdmytro@gmail.com (D.R.); senenkoanton@gmail.com (A.S.); 7 Department of Biology and Chemistry, Belgorod State University, 85 Pobedy Str., 308015 Belgorod, Russia; max_evstigneev@mail.ru; 8 Institute of Chemistry and Biotechnology, Technical University of Ilmenau, 25 Weimarer Str., 98693 Ilmenau, Germany; uwe.ritter@tu-ilmenau.de; 9 Intercollegiate Faculty of Biotechnology, UG-MUG (University of Gdansk and Medical University of Gdansk), Abrahama 58, 80-307 Gdańsk, Poland

**Keywords:** Berberine, C_60_ fullerene, nanocomplex, lung cancer cells, Lewis lung carcinoma, metastasis in vivo

## Abstract

Effective targeting of metastasis is considered the main problem in cancer therapy. The development of herbal alkaloid Berberine (Ber)-based anticancer drugs is limited due to Ber’ low effective concentration, poor membrane permeability, and short plasma half-life. To overcome these limitations, we used Ber noncovalently bound to C_60_ fullerene (C_60_). The complexation between C_60_ and Ber molecules was evidenced with computer simulation. The aim of the present study was to estimate the effect of the free Ber and C_60_-Ber nanocomplex in a low Ber equivalent concentration on Lewis lung carcinoma cells (LLC) invasion potential, expression of epithelial-to-mesenchymal transition (EMT) markers in vitro, and the ability of cancer cells to form distant lung metastases in vivo in a mice model of LLC. It was shown that in contrast to free Ber its nanocomplex with C_60_ demonstrated significantly higher efficiency to suppress invasion potential, to downregulate the level of EMT-inducing transcription factors SNAI1, ZEB1, and TWIST1, to unblock expression of epithelial marker E-cadherin, and to repress cancer stem cells-like markers. More importantly, a relatively low dose of C_60_-Ber nanocomplex was able to suppress lung metastasis in vivo. These findings indicated that сomplexation of natural alkaloid Ber with C_60_ can be used as an additional therapeutic strategy against aggressive lung cancer.

## 1. Introduction

Plant alkaloids are the natural source of pharmacologically active agents, which have been widely used for years in traditional Eastern medicine. The representative of alkaloids Berberine [5,6-dihydro-9,10-dimethoxybenzo (g)-1,3-benzodioxolo (5,6-a) quinolizinium] (Ber) is an isoquinoline, which belongs to the structural class of protoberberines. Ber is present in the roots, rhizome, and stems of Berberis species and was isolated from a variety of Chinese herbs. It possesses a variety of therapeutic activities such as antibacterial, antiviral, anti-inflammatory, antidiabetic and has the potential to treat numerous diseases [1,2]. The polypharmacological mode of Ber action is explained by the presence in its molecule of the positive quaternary amine group, which interacts with nucleophilic and anionic moieties in the structure of biomolecules involved in multiple cell’ regulatory pathways [3,4]. Ber’ multi-specificity and belonging to the old traditional medicines do not indicate its disadvantage as an anticancer drug because many synthetic drugs are also not monospecific. Besides, there is no need to evaluate Ber toxicity and pharmacokinetics, since they have already been determined in animal models and humans [5,6]. It is believed that Ber has the potential for repurposing in therapy, that is, being an already approved drug against noncancer diseases, it can be recommended for cancer treatment [7,8]. 

In recent years, the antitumor activity of Ber has attracted the special attention of scientists, as promising results have been obtained against various cancers [9,10]. However, the development of Ber-based anticancer drugs is limited for several reasons. Ber is known for its low effective concentration, poor membrane permeability, limited bioavailability, and short plasma half-life resulting from its weak absorption, efflux by intestinal permeability-glycoprotein, and extensive metabolism in the liver [11,12]. These obstacles can be overcome with the help of rapidly developing biotechnological approaches with new formulations based on the use of nanostructures for the efficient delivery of anticancer drugs into cells [13]. Design and testing of nanoparticulated Ber delivery systems with the use of lipids [14], silver [15], zinc oxide [16], chitosan [17], and dendrimer [18] nanoparticles become a hot topic in experimental research. Computer-aided molecular tools, in particular, molecular dynamics (MD) simulation [19] are recognized to be useful both for understanding drug interaction with nanoparticles and optimization of drug delivery vehicles design.

The ability of biocompatible and bioavailable representative of carbon nanostructures C_60_ fullerene (C_60_) to be covalently or noncovalently functionalized with bioactive molecules and therapeutics makes this nanostructure a promising drug carrier [20,21].

We have previously demonstrated that C_60_ could be a favorable nanoplatform for targeted delivery of chemotherapeutic drugs and optimization of their efficiency against cancer cells. We have developed a quick and simple strategy for the non-covalent complexation of Doxorubicin (Dox), Cisplatin (Cis), and Ber with C_60_ and confirmed its efficiency for the substantial potentiation of drugs intracellular uptake and their cytotoxic effects in the range of low doses against leukemic and lung cancer cell lines [22,23,24].

It should be considered that many compounds cytotoxic in vitro do not inhibit tumor growth in vivo. Therefore, the antitumor effects of Ber in the form of nanocomplexes still require further study in living organisms. Previously, using a mouse model of Lewis lung carcinoma (LLC), we demonstrated a therapeutic potency of C_60_-Ber nanocomplexes to delay and suppress tumor growth, while no anticancer effect of the free Ber in the equivalent 7.5 mg/kg dose in the group of tumor-bearing animals was detected [25].

Another problem is that in most cases, cancer mortality is caused not by the primary tumor but by its metastasis into distant organs. Since about 90% of all cancer deaths are associated with tumor metastasis, and the high frequency of metastases is still an obstacle to achieve satisfying treatment [26,27], the development of drugs to suppress the invasiveness and metastatic capacity of cancer cells remain the focus in cancer research. 

To gain an invasive phenotype, cancer cells undergo epithelial-mesenchymal transition (EMT), whereby cells lose markers of epithelial phenotypes, such as E-cadherin and other molecules involved in the formation of intercellular contacts, and upregulate mesenchymal markers, such as vimentin, to enhance mobility and dissemination. At the molecular level, EMT is triggered by the expression of key transcription factors of ZEB, SNAIL, and TWIST families [28,29]. Since EMT is a determining factor in tumor progression, its inhibition could be a promising therapeutic strategy for treating highly invasive lung cancer.

Taking into account the above, the aim of this study was to assess the effect of both free Ber and C_60_-Ber nanocomplex in low concentrations on LLC cells invasion potential, expression of EMT transcription factors and key protein markers in vitro and to evaluate the potential of studied preparations to overcome cancer lung metastasis in vivo in a mice model of LLC.

## 2. Materials and Methods 

### 2.1. Structure Modeling of C_60_-Ber Nanocomplex 

The methodology of energy decomposition for aromatic complexes, reported in [30] was used for estimation of C_60_ complexation with Ber molecule. 

The spatial structures of the nanocomplexes of C_60_ with different number of Ber molecules were calculated by the methods of molecular mechanics (Charmm 27 force field) using the HyperChem program (Hypercube, Toronto, Ontario, Canada). Modeling of the aqueous environment was performed by placing water molecules in the form of TIP3P into a cubic box with side length of 4 nm. Optimization geometry of the C_60_-Ber nanocomplexes with different number of Ber molecules was carried out by means of potential energy minimization using the steepest descent method. 

MD simulation of C_60_ with different numbers of Ber molecules was accomplished with the help of the Hyperchem program. We used the following parameter setup in the calculations: run time 1 ns, step size 0.001 ps, constant temperature 300 K, bath relaxation time 0.1 ps.

### 2.2. Preparation of C_60_-Ber Nanocomplex

The highly stable pristine C_60_ aqueous colloid solution (2.6 mg/mL, purity > 99.5%,) was prepared as described in [31,32]. Ber was dissolved in a distilled water at initial concentration of 1 mg/mL. C_60_ and Ber solutions were mixed in a 2:1 C_60_-Ber molar ratio (208:104 µM), and the formation of a stable noncovalent nanocomplex was confirmed by the UV–Vis spectroscopy, atomic force microscopy (AFM, Solver Pro M system, NT-MDT, Moscow, Russia), and dynamic light scattering (DLS) measurements as described in [24]. 

### 2.3. Cell Culture

LLC cells were kindly supplied by the Bank of Cell Cultures and Transplantable Experimental Tumors of R.E. Kavetsky Institute of Experimental Pathology, Oncology, and Radiobiology, NAS of Ukraine (Kyiv, Ukraine). Cells were maintained in DMEM medium, supplemented with 10% FBS, 50 U/mL penicillin, and 100 μg/mL streptomycin (Gibco^®^, Amarillo, TX, USA) at 37 °C and 5% CO_2_ in a humidified atmosphere. The cells were split 1:3–1:5 every 2–3 days at 70–80% confluency. Treatments with 0.25% Trypsin-EDTA (Thermo Fisher Scientific, Waltham, MA, USA) or 2 mM EDTA (Sigma-Aldrich, St. Louis, MI, USA) were used to detach adherent cells. 

### 2.4. In Vitro Invasion Assay

The effect of C_60_-Ber nanocomplex on LLC cells invasion was analyzed by modified Boyden chamber assay. Briefly, LLC cells were detached with 2 mM EDTA, and 3 × 10^4^ cells were seeded into the upper chamber of Matrigel-coated Transwells (Greiner Bio-One, Kremsmünster, Austria) in serum-free DMEM medium. The lower chamber contained a complete DMEM medium. Cells were treated with C_60_ (20 μM), Ber (5 or 10 μM), or 2:1 C_60_-Ber nanocomplex (5 or 10 μM Ber-equivalent) for 24 h. Then non-migrated cells were removed with cotton swab, and migrated cells were fixed in 4% PFA and stained with 0.1% crystal violet. Migrated cells were visualized using an inverted microscope (Olympus Corporation, Tokyo, Japan) at magnification ×200, and photographed. The average number of cells per view field was used for invasion quantification.

### 2.5. Western Blot Analysis

LLC cells were seeded into 100 mm Petri dish and incubated for 24 h (70–80% confluency). Cells were treated with C_60_ and Ber separately or C_60_-Ber nanocomplexes in 10 µM Ber-equivalent concentration for 24 h. Then, protein lysates were prepared as described previously [33]. A total of 30 μg of protein was separated on 10% polyacrylamide gel and transferred to nitrocellulose membrane. Primary antibodies (monoclonal anti-E-cadherin (Cell Signaling, Cell Signaling, Danvers, MA, USA), anti-vimentin (Sigma-Aldrich, St. Louis, MI, USA), anti-β-actin antibodies (Sigma-Aldrich, St. Louis, MI, USA), and polyclonal anti-Ruk/CIN85 antibody [34]) and corresponding secondary HRP-conjugated antibodies (Sigma-Aldrich, St. Louis, MI, USA) were used for Western-blot analysis. The enhanced chemiluminescence kit (Thermo Fisher Scientific, Waltham, MA, USA) was used for detection. Semi-quantitative analysis was performed by densitometry using Gel-Pro Analyzer 3.0 (Media Cybernetics, L.P., Rockville, MD, USA).

### 2.6. Quantitative PCR Analysis

LLC cells were seeded into 60 mm Petri dish and incubated for 24 h (70–80% confluency), then treated with studied compounds for another 24 h. Total RNA was isolated using a commercial column-based Direct-zol RNA kit (Zymo Research, Irvine, CA, USA) according to the manufacturer’s recommendations. Commercial kit M09 (Ukrainian Genetic Technologies, Kyiv, Ukraine) was used for cDNA synthesis from 1 µg of total RNA per reaction. Real-time PCR was performed on qTOWER 2.0 thermal cycler (Analytik Jena, Jena, Germany) with SYBR Green-based kit M02 (Ukrainian Genetic Technologies, Kyiv, Ukraine). B2m was used for normalization, and ΔΔCt method—for quantification. Primers sequences are presented in Table 1.

### 2.7. In Vivo Metastatic Growth Study

LLC cells (5 × 10^5^) in 100 μL of saline were injected into the axilla of the right forelimb of C57Bl male mice aged 6 weeks. All experiments were carried out according to the recommendations of the European Convention for the Protection of Vertebrate Animals used for Research and Scientific Purposes and the Procedure for conducting animal research, experiments by scientific institutions (Order N249 of the Ministry of Education and Science of Ukraine from 1 March 2012) and were approved by the ethical committee of Palladin Institute of Biochemistry of NAS of Ukraine. The mice were held under standard laboratory conditions and had free access to laboratory rodent chow and water.

On the second day after cells inoculation, the mice were randomized into 4 treatment groups of n = 7 mice each, and 200 µL of the following agents were injected intraperitoneally (i.p.) 5 times every other day: 1—0.9% saline (control, untreated); 2—C_60_ in a total dose 15 mg C_60_/kg; 3—Ber in a total dose 7.5 mg/kg; 4—С_60_-Ber nanocomplex in a total dose 22.5 mg/kg (15 mg C_60_/kg and 7.5 mg Ber/kg). 

At day 29 of the experiment, the mice were sacrificed by the standard procedure of cervical dislocation. Lungs were isolated, weighed, and fixed in Bouin’s solution. Metastatic nodules on the surface of the lungs were counted under light microscope. Lung tissues were fixed in 10% formalin and embedded in paraffin. Paraffin sections (5 μm) were stained with hematoxylin-eosin (H&E, Global Biomarketing Group, Kyiv, Ukraine) for histological evaluation using the Olympus microscope (CKX53, Olympus, Shinjuku, Tokyo, Japan). 

### 2.8. Statistics

All experiments were carried out with a minimum of 3 replicates. Data were presented as mean ± SEM. Statistical analysis was performed using Origin 9.0 software (OriginLab Corporation, Northampton, MA, USA) by one-way ANOVA followed by Fisher LSD post-hoc test. Differences between groups were considered to be significant at *p* < 0.05.

## 3. Results

### 3.1. Calculation

Using the method of energy decomposition suggested in [30], we calculated the energy terms composing the net free energy (Δ*G*_total_) of C_60_ complexation with one Ber molecule (Table 2). Δ*G*_total_ was summarized from the contributions to the net energy from physico-chemical sources of different nature: Δ*G*_total_ = Δ*G*_vdw_ + Δ*G*_el_ + Δ*G*_hydr_ + Δ*G*_H-bonds_ + Δ*G*_entr_, where the subscript indexes of Δ*G* denote the contribution to the net energy from van-der-Waals, electrostatic, hydrophobic forces, hydrogen bonds, and factors originated from changes in degrees of freedom predominantly of an entropic nature, respectively. For some terms (Δ*G*_vdw_, Δ*G*_el_, Δ*G*_H-bonds_) the computation of the free energy was conducted separately in a vacuum and in water (intermolecular—im and solvation*—*solv, respectively, see Table 2). The entropic term (Δ*G*_entr_) was the sum of the three main components: Δ*G*_transl_, Δ*G*_rot_, and Δ*G*_vib_, which denote the changes in free energies of translational, rotational, and vibrational degrees of freedom upon complexation, respectively. The total vibrational contribution to the energy of complexation consisted of two terms related to change in vibrations of chemical bonds (Δ*G*_vib(I)_), and mechanical oscillations (Δ*G*_vib(II)_)_._

Analysis of the calculated energies related to various physical factors (i.e., van-der-Waals, electrostatic, hydrophobic forces) well agrees with previous estimation [24]. As one can see, stabilization of C_60_-Ber nanocomplex comes predominantly from van-der-Waals intermolecular interaction (Δ*G*_im_) and, to a lesser extent, hydrophobic interaction (ΔG_hydr_). The van-der-Waals interaction with solvent (Δ*G*_solv_) was unfavorable. The energies corresponding to the loss of translational (Δ*G*_tr_) and rotational (Δ*G*_rot_) degrees of freedom were unfavorable, whereas the creation of new vibrational modes of chemical bonds (Δ*G*_vib(I)_) and mechanical oscillation (Δ*G*_vib(II)_) were favorable. All types of electrostatic interactions do not contribute to the stability of this nanocomplex. The estimated value of Δ*G*_total_ obtained by summation of calculated component energies well matches the experimental one (Δ*G*_exp_) reported before [24], which confirms the appropriateness of the method used.

In order to estimate the maximal adsorption ability of one C_60_ molecule to bind Ber, MD simulation was carried out for various potential nanocomplexes, i.e., C_60_ with n = 1, 2,…, 7 Ber molecules. It was found that the maximal number of Ber molecules, which can interact with the C_60_ surface directly, equals 5. In the case of nanocomplexes with more than 5 Ber molecules, we noted the increased overlap (interaction) of Ber chromophores resulted in stacking them one above the other, which destabilizes the nanocomplex.

**Figure 1:**
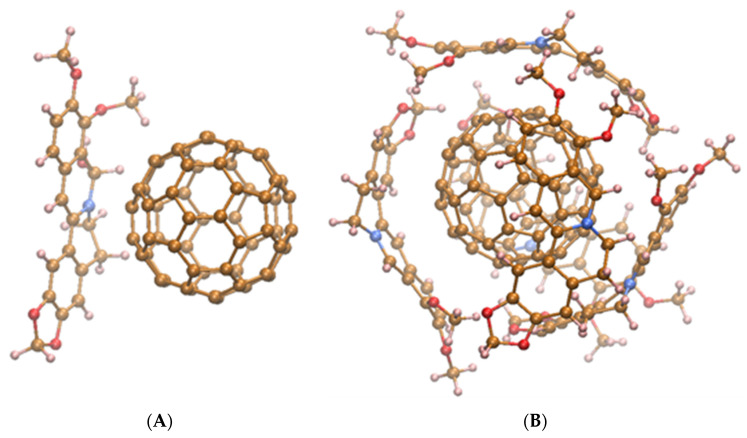
The energy-minimized structure of nanocomplexes formed at C_60_ binding with one (**A**) or five (**B**) Ber molecules.

Figure 1 demonstrates the energy-minimized structure of nanocomplexes formed at C_60_ noncovalent binding with one (corresponds to С_60_-Ber at ~2:1 molar ratio) or five (corresponds to С_60_-Ber at ~1:2 molar ratio) Ber molecules.

Since according to our previous data, the cytotoxicity of С_60_-Ber nanocomplexes at different molar ratios against leukemic and lung cancer cells following the order: free Ber < 1:2 < 1:1 < 2:1 [25,28] in this study, we used 2:1 C_60_-Ber nanocomplex.

### 3.2. C_60_-Ber Nanocomplex Demonstrates the High Ability to Suppress the Invasion Potential of LLC Cells In Vitro

**Figure 2:**
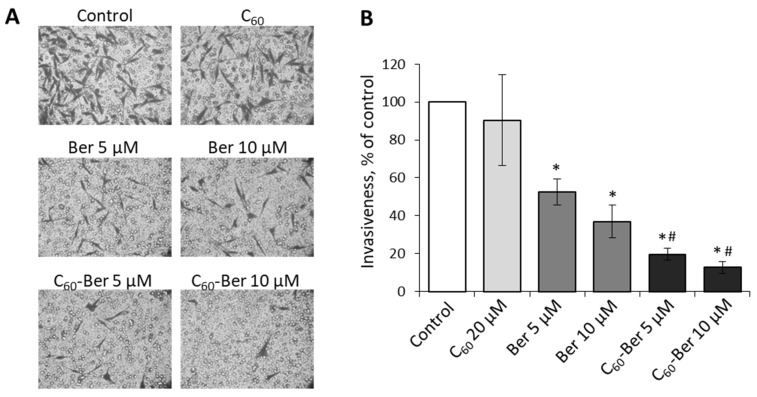
LLC cells invasion is inhibited at 24 h treatment with Ber or C_60_-Ber nanocomplex in a low concentration: (**A**)—representative micrographs of invasive cells migrated in Boyden chamber assay; (**B**)—quantitative analysis of cell invasion (100% was taken for untreated cells). * *p* < 0.05 compared to control; # *p* < 0.05 compared to free Ber.

Lung cancer cells are highly aggressive and able to detach from the primary tumor, invade through the extracellular matrix with rapid dissemination and intravasation into secondary organs, followed by initiation of proliferation at the metastatic site. To estimate Ber effect on the invasive potential of LLC cells in vitro, we used Boyden chamber matrigel assay that allows quantifying invasive cells that migrated through the reconstituted basement membrane (Figure 2). Our previous study demonstrated that at low (5 and 10 µM) concentrations, free Ber has no effect on LLC viability at 24 h incubation, while treatment with 2:1 C_60_-Ber nanocomplexes at Ber equivalent concentrations was followed by the decrease of viability approximately by 50% as compared to the control untreated cells [25]. 

The data obtained allow us to draw the following conclusions. As it turned out, the capability of C_60_-Ber nanocomplex to inhibit the viability and invasion potential of aggressive lung cancer cells in vitro was higher than that of free Ber. These results also indicate that Ber-induced suppression of LLC cell invasion was not only linked to cell death but could be determined by alkaloid interference into signaling pathways involved in the control of EMT.

### 3.3. C_60_-Ber Nanocomplex Effectively Modulates the Expression of EMT Effectors

**Figure 3:**
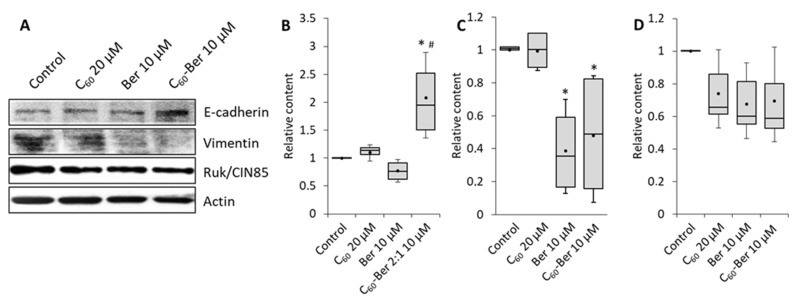
C_60_-Ber nanocomplex modulates the content of EMT molecular markers in LLC cells: (**A**)—representative results of Western-blot analysis; (**B**–**D**)—quantitative analysis of E-cadherin, vimentin, and Ruk/CIN85 content, respectively. Box-plot legend: box represents second and third quartiles; whiskers—minimal and maximal values; dot—mean value. * *p* < 0.05 compared to control; # *p* < 0.05 compared to free Ber.

To clarify the molecular mechanisms by which Ber represses the invasion potential of LLC cells we studied the expression of EMT key effectors with the use of Western blotting. Cells were treated with 10 µM Ber or Ber nanocomplex for 24 h, and the content of adhesion protein E-cadherin as the marker of epithelial cell phenotype, intermediate filaments protein vimentin as a mesenchymal marker, and adaptor protein Ruk/CIN85 as the indicator of increased malignant properties [33] was estimated (Figure 3).

Representative results of Western-blot (Figure 3A) and densitometry analysis of proteins relative content (Figure 3B–D) showed that 10 µM C_60_-Ber nanocomplex modulate the protein content of both epithelial and mesenchymal markers in LLC cells. Importantly, no reliable effect of free 10 µM Ber on E-cadherin expression was detected, while a strong increase was observed after the treatment with C_60_-Ber nanocomplex (Figure 3B). Vimentin content was found to be decreased by both free Ber and, to a greater extent, by C_60_-Ber nanocomplex (Figure 3С). At the same time, Ruk/CIN85 content had only a tendency to decrease after the treatment with free Ber and C_60_-Ber (Figure 3D). No effect of free C_60_ in a concentration equivalent to those in nanocomplex on studied parameters was detected. These data allow us to suggest that C_60_-Ber nanocomplex can induce lung cancer cells to transition from mesenchymal to epithelial phenotype by enhancing E-сadherin expression and suppressing vimentin expression.

### 3.4. Expression of EMT Related Transcription Factors and Cancer Stem Cell Surface Markers Is Suppressed by C_60_-Ber Nanocomplex

**Figure 4:**
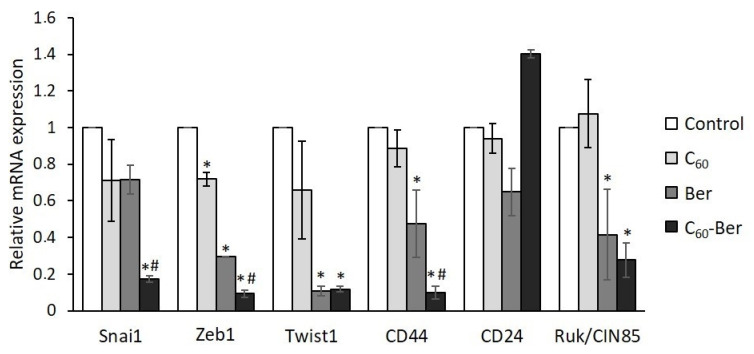
C_60_-Ber nanocomplex effectively attenuates the expression of EMT and CSC molecular markers in LLC cells. * *p* < 0.05 compared to control; # *p* < 0.05 compared to free Ber.

The reverse cellular transdifferentiation process from epithelial to mesenchymal states and vice versa is known to be a dynamic process driven by a number of EMT transcription factors depending on cellular context. To reveal the possible molecular mechanisms of C_60_-Ber involvement in the regulation of E-cadherin content in LLC cells we used qPCR and examined the levels of mRNA expression of three transcription factors, which are the key interdependent EMT regulators and inducers of mesenchymal phenotype, in particular, zinc-finger binding factors SNAI1 and ZEB1, and basic helix–loop–helix factor TWIST1. SNAI1 and ZEB1 are known to downregulate E-cadherin expression by binding to the elements of E-cadherin promotor, while TWIST1 induced suppression of E-cadherin expression by activation of SNAI1 transcription [35,36]. As can be seen from Figure 4, Ber alone suppressed ZEB1 and TWIST1 expression with no effect on SNAI1 expression as compared to control values, whereas C_60_-Ber nanocomplex significantly and with equal efficiency up to 10–15% of the control level suppressed the expression of all three transcription factors. 

The increased expression of SNAI1, TWIST, and ZEB1 is thought to be associated with the acquisition of the cancer stem cell (CSC) phenotype, high metastatic potential, and increased resistance to chemo- and radiotherapy [37,38]. To determine whether Ber-induced down-regulation of these transcription factors affects LLC cells phenotype, we examined the expression of CD44 and CD24 cell surface markers, considering that CD44^+^/CD24^-^ phenotype is associated with stemness. Treatment with Ber reduced the expression of CD44 with no reliable effect on the level of CD24 transcripts, while treatment with C_60_-Ber nanocomplex led to almost complete down-regulation of CD44 and up-regulation of CD24 transcription (Figure 4), thus reducing the proportion of highly migratory LLC cells with stem cell phenotype. Moreover, both Ber and C_60_-Ber nanocomplex treatment significantly decreased expression of adaptor protein Ruk/CIN85 (Figure 4), which was found to modulate CD44 and CD24 expression levels and to be involved in the maintenance of CSC features in breast cancer cells [39]. 

Taken together, these results suggest that LLC cells treatment with C_60_-Ber nanocomplex allows downregulating the level of EMT-inducing transcription factors SNAI1, ZEB1, and TWIST1, to unblock expression of invasion suppressor E-cadherin and to repress CSC-like characteristics.

### 3.5. C_60_-Ber Nanocomplex Effectively Suppresses LLC Cells Metastasis to Lung 

**Figure 5:**
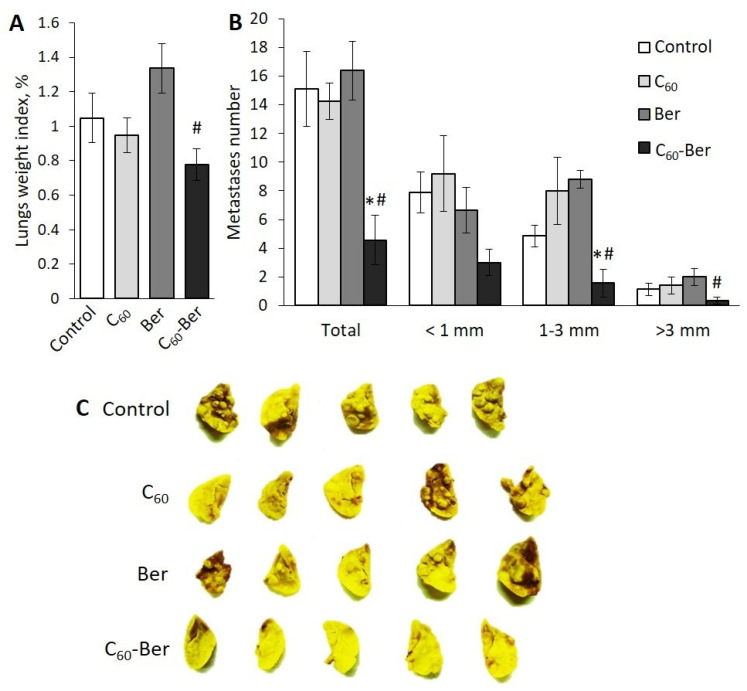
С_60_-Ber nanocomplex effectively suppresses metastasis of LLC cells in vivo: (**A**)—lung weight index at day 29 after LLC cells inoculation; (**B**)—the number of metastases of different size in lung tissue; (**C**)—representative photographs of lungs. * *p* < 0.05 compared to control; # *p* < 0.05 compared to free Ber.

At the next stage of our work, we used the LLC mouse model to evaluate the effect of tumor-bearing mice treatment with C_60_-Ber nanocomplex on metastasis of tumor cells to the lung. On day 29, after LLC cells inoculation, the lungs weight index in the groups of tumor-bearing mice treated with С_60_, free Ber, or С_60_-Ber nanocomplex did not differ significantly compared to that in the control group of untreated mice (Figure 5A).

It was found that the lungs of mice in control, C_60_, and Ber groups had a bumpy appearance, and the degree of metastasis was evident by the formation of numerous metastatic lesions clearly visible on the lung surface (Figure 5C). In contrast, the lungs of mice in С_60_-Ber nanocomplex group had a smooth and uniform surface and contained only single metastatic lesions or did not contain any. We monitored the number of lung metastases of different sizes and found out that its total number in the C_60_-Ber nanocomplex group was on average more than three times less than in the control group due to a significantly reduced number of metastases with a size of 1–3 and >3 mm (Figure 5B).

These data confirm that C_60_-Ber nanocomplex injected in a low Ber-equivalent concentration (7.5 mg/kg) can effectively inhibit the ability of tumor cells to form distant lung metastases.

**Figure 6:**
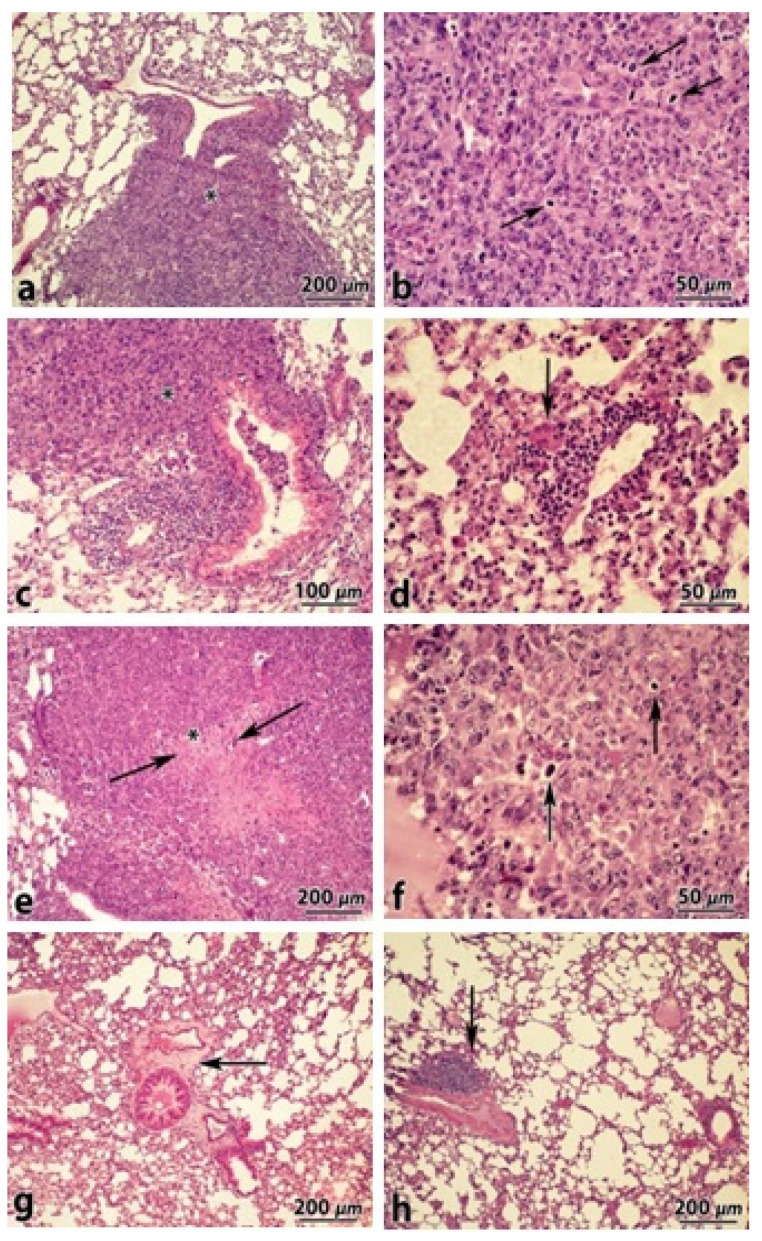
Histological examinations of H&E stained lung tissues sections from the mice of control (**a**,**b**), C_60_—(**c**,**d**), Ber—(**e**,**f**), and C_60_-Ber nanocomplex—(**g**,**h**) treated groups. Arrows indicate metastatic nodules (in **d**–**f**) and lymphoid infiltrates (in **g**,**h**).

Histological examination of H&E-stained lung tissue sections revealed the presence of metastatic infiltration under the pleura, around the bronchi and blood vessels with foci of necrosis in the lungs of mice of the control, C_60_ and Ber groups. Cytomorphological characteristics such as cells atypia and polymorphism, a large number of mitoses (Figure 6a–f) confirmed that these clusters corresponded to metastases of primary LLC, that actively replaced the lung tissue. It was noted that in the lungs of mice of the C_60_ group, the “protective sleeve” around the bronchi and blood vessels consisted of numerous lymphocytes, single macrophages, and giant multinucleated cells was formed (Figure 6d). In the lung tissue of mice of the C_60_-Ber nanocomplex group, similar infiltrates were detected (Figure 6g,h), which suggests that their formation was associated with C_60_ involvement in the antitumor immune response.

## 4. Discussion

Suppression of metastasis is considered to be the main problem in cancer therapy, and in recent years, Ber attracted the attention of researchers for its antimetastatic activity. Given the relatively slow and weak action of Ber, it was usually used in the range of 10–100 µM concentrations and 24 h incubation of cancer cell lines of different tissue origins [40,41,42]. In this study, we have shown that the complexation of Ber with C_60_ significantly potentiates Ber ability to inhibit LLC cells’ invasiveness in vitro in a low 10 µM concentration. We attributed this effect to С_60_ ability to enhance Ber intracellular uptake since no effect of C_60_ alone on cells invasion was detected. Taking into account the observed effect of C_60_-Ber nanocomplex on the expression levels of regulatory proteins associated with EMT, we suggested that C_60_-Ber nanocomplex contributes to the stabilization of low-invasive epithelial phenotype of aggressive LLC cells through inducing EMT. The data obtained showed that in contrast to 10 µM free Ber C_60_-Ber nanocomplex in Ber-equivalent concentration was able to enhance E-cadherin expression. This can be explained by our findings that C_60_-Ber nanocomplex much more effectively than free Ber targeted EMT-associated transcription factors SNAI1 and ZEB1 and downregulated their expression.

Recent research advancements suggest that invading carcinoma cells, which function as migrating CSC, have undergone an EMT process and that circulating tumor cells can be discharged into the blood circulation much sooner than tumor metastasis appeared [43]. In this regard, our data concerning the potential of C_60_-Ber nanocomplex to repress CSC-like characteristics of LLC cells appear to be important. It was shown that treatment with free Ber was followed by negative modulation of CD44 with no effect on CD24 expression whereas treatment with C_60_-Ber nanocomplex led to almost complete down-regulation of CD44 and up-regulation of CD24 transcripts suggesting the ability of C_60_-Ber nanocomplex to reduce the content of migratory LLC cells with high metastatic potential. 

The advantages of using drugs nanoparticulated forms to inhibit cancer cells proliferation are now widely discussed, but the perspective of their application as anticancer preparations for clinical treatment needs to be approved in vivo. Despite the fact that nanoscaled size allows nanoparticles to evade the reticuloendothelial system by escaping macrophage uptake, to extend the half-life, and to use the EPR effect for passive penetration into the tumor, their behavior in the body is still poorly understood. Analysis of the reported accumulation of nanoparticles in preclinical tumor models revealed that the percentage of administered nanoparticles that can reach the targeted tumor achieves 0.7% of injected dose [44]. In this regard, it is important that in contrast to such inert components of nanoparticulated systems as metals or liposomes, C_60_ and its derivatives can not only be a platform for drug delivery, but also possesses biological activity and can be involved in cellular regulatory processes by exhibiting ROS scavenging, anti-inflammatory and immunomodulatory effects [21,45,46,47]. We suggest that inhibition of spontaneous pulmonary metastases by C_60_-Ber nanocomplex, which we proved in the LLC model, could be explained not only by suppression of LLC cells invasiveness and dissemination but also by C_60_ immunomodulatory effects in blood circulation and tumor microenvironment. The finding of infiltrates with high lymphocyte densities in lung tissue of tumor-bearing mice treated with C_60_ and C_60_-Ber nanocomplex obviously indicates the up-regulation of the immune anticancer response and may testify a favorable outcome due to immunosurveillance [48]. 

In our previous studies, it was shown that when Dox or Cis, which are the golden standards of anticancer medicines, were immobilized on C_60_, their intracellular concentration in cancer cells was increased, leading to higher toxicity and a more pronounced antitumor effect compared with the free drug against various human tumor cell lines in vitro [22,23,49,50] and metastatic mice LLC in vivo [51,52]. This work shows that Ber, immobilized on C_60_, almost completely blocks metastasis in mice.

## 5. Conclusions 

Taken together, our findings demonstrated that noncovalent C_60_-Ber nanocomplex inhibited LLC cells invasion in vitro by reversion of EMT and repression of CSC-like characteristics. The stronger antimetastatic potency of C_60_-Ber nanocomplex compared to that of free Ber in a low equivalent concentration was confirmed in vivo by significant suppression of LLC cells lung metastasis and induction of the immune anticancer response. The results obtained showed that the complexation of the natural alkaloid Ber with the C_60_ can be an additional therapeutic strategy in the complex treatment of metastatic lung cancer.

## Figures and Tables

**Table 1:** Primers sequences, used for real-time PCR.

Gene	Forward Primer	Reverse Primer
Snai1	TCTGAAGATGCACATCCGAAGCCA	AGGAGAATGGCTTCTCACCAGTGT
Zeb1	GGAGGAGGTGACTCGAGCATTTAG	TAATACTGTCTGGTCTGCTGGC
Twist1	CTCAGCTACGCCTTCTCCGT	CCTCTGGGAATCTCTGTCCAC
Cd44	AGAGCACCCCAGAAAGCTAC	GTAGTTGCACTCGTTGTGGG
Cd24	GCGAGCTTAGCAGATCTCCAC	CGGTGCAACAGATGTTTGGT
Sh3kbp1 (Ruk/CIN85)	CGCCAACTTTCACGCTGCTT	TGACCTCACCCACGCTGATT
B2m	ACCGTCTACTGGGATCGAGA	TGCTATTTCTTTCTGCGTGCAT

**Table 2:** Energy terms (ΔG, kcal/mol) of C_60_ complexation with Ber molecule.

Nano-Complex	Δ*G*_entr_				Δ*G*_el_		Δ*G*_vdw_		Δ*G*_H-bonds_	Δ*G*_hydr_	Δ*G*_total_	Δ*G*_exp_
	Δ*G*_tr_	Δ*G*_rot_	Δ*G*_vib(I)_	Δ*G*_vib(II)_	Δ*G*_solv_	Δ*G*_im_	Δ*G*_vdw__(solv)_	Δ*G*_vdw__(im)_				
C_60_-Ber	9.8	8.4	−6.0	−9.5	0.0	0.0	16.8	−19.0	−1.8	−10.1	−11.3	−6.0 [24]
**Σ**	2.8				0.0		−2.2					

## Data Availability

Data sharing not applicable.
